# Investigating the role of Arabic language in sustaining socio-cultural identity and family values in Emirati society

**DOI:** 10.3389/fsoc.2025.1641732

**Published:** 2025-08-01

**Authors:** Bilal Zakarneh, Diana Amin Mohammad Mahmoud

**Affiliations:** ^1^Department of Languages and Cultures, Ajman University, Ajman, United Arab Emirates; ^2^Department of Mathematics, Amman Arab University, Amman, Jordan

**Keywords:** Arabic language, socio-cultural identity, family values, cultural transmission theory, United Arab Emirates

## Abstract

Language is a critical social and cultural identity component, further affecting family values and cohesion. Particularly, the importance of the Arabic language (UAE) cannot be overstated due to its distinct role in positioning the United Arab Emirates as a culturally distinct society. Considering the importance of the Arabic language, this study also examined its role and effect on socio-cultural identity and family values in Emirati society. Based on the proposition of Cultural Transmission Theory, this study gathered data from 200 young individuals currently residing in Ajman, UAE. Results revealed a positive effect of the Arabic language on socio-cultural identity, implying that it helps recognize local values and social participation and helps with the significance of social laws. The effect of the Arabic language on family values also remained significant, suggesting that it improves family relations, helps understand values and a sense of respect, and strengthens relationships with ancestral heritage. Overall, these results indicated the Arabic language's strong, sportive role in enhancing the sense of commitment and encouraging humility in society. These results also showed that the role of the Arabic language in promoting a sense of unity and social stability further leads to improved social participation. Thus, the role of the Arabic language is acknowledged and highlighted, further emphasizing the role of language as a source of communication and socio-cultural cohesion. Finally, study implications and limitations are discussed.

## 1 Introduction

Language plays a crucial role in promoting and preserving national and cultural identity. Maintaining cultural heritage is important for social cohesion, as communication in the native language stabilizes identity and a sense of belonging (Hovy and Yang, 2021). Language also enriches society by enabling the exchange of ideas, perspectives, and traditions. According to Schwartz and Cieciuch (2022), language acts as a vehicle for passing down knowledge and wisdom across generations. It is a source of preserving mythology, customs, and historical narratives, ensuring that a nation's collective memory endures. Written and oral traditions rooted in language contribute to the preservation of distinct cultural and ethnic identities. According to Durmus et al. (2024), the role of language in promoting equitable participation and inclusivity cannot be denied. In this regard, language also helps communicate with the public services, enabling the organizations to ensure equal access to resources among the masses without discrimination. Language strengthens a society to a better position to engage in trade and diplomacy, cultural and social exchange, improving intercultural relationships and participation in national and international affairs (Masoud et al., 2024).

Similarly, the United Arab Emirates is a prime example of cultural and social cohesion, enriched by diversity and national affiliation (Siemund, 2019). Despite the United Arab Emirates (UAE) population from multiple racial, national, and ethnic backgrounds, this diversity is enriched by the Arabic language as known and spoken countrywide (Awad et al., 2021). According to Baycar (2023), the UAE officially promotes an Arab and Islamic Emirati identity that includes non-Emirati individuals. Shared social spaces between local Emiratis and expatriates highlight the nation's social and cultural diversity. In this context, the Arabic language serves as an important medium and a bond between diverse populations and national social and cultural identities, reflecting the background enriched by language. This inclusivity has further contributed to improving and sustaining the UAE's social progress, traditions, and cultural norms (Elnagar et al., 2021). Besides, this progress is equally acknowledged by the young generation in the United Arab Emirates, who not only consider Arabic as a part of their national identity but also as a source of their positive cultural representation, stability of their family norms and cultural enhancement (Elnagar et al., 2021). The problem addressed in this research comprises the role of the Arabic language in the social and cultural scenario of the United Arab Emirates. Particularly, its effect on promoting Socio-Cultural and Family Values is examined. Derakhshan et al. (2024) argued that language reflects cultural identity and passes knowledge down through generations. It ensures inclusivity by granting everyone opportunities and access to resources regardless of racial, national, and ethnic characteristics. Besides, language also removes a nation's international identity by facilitating deeper relationships with the global community.

Considering the importance of the Arabic language in UAE's society, this study aims to examine its effects on broad social and cultural systems. Its role in affecting the perceptions of the young generation toward socio-cultural identity and family systems is especially important. Notably, this research fills an important gap as Emirati society has transitioned largely due to technological transformation during COVID-19 (AbuSamra and Zamoum, 2025; Alsalhi et al., 2022; Lutfi et al., 2021; Vally et al., 2023). The use of technology has increased, leading to robust exposure to other cultures, languages, and societies, which may further affect the perceptions of the young Emirati population about the social and cultural system. Considering this gap, this research provides practical and real-time considerations for the readers, future researchers, and other stakeholders regarding the Arabic language, promoting socio-cultural identity and stabilizing the family system.

## 2 Review of literature

### 2.1 Role of language in supporting socio-cultural identity

Socio-cultural identity plays a critical role in shaping the complexity of social interactions within societies (Glăveanu and Tanggaard, 2014). Identity is a rigorous construct affected by contextual factors and cultural shifts rather than a fixed trait. Concerning language and socio-cultural identity. It transfers a shared sense of belonging (Zubareva, 2020), mutual understanding and how individuals or groups perceive themselves within an evolving societal landscape. Accordingly, existing literature (Aziz, 2025; Bucholtz and Hall, 2005) highlights the role of language in constructing and sustaining cultural identity, showing how people naturally classify themselves and others based on certain cultural characteristics. This process has become increasingly complex, especially in the current era of digitalization and social communication, as individuals manage multiple overlapping identities and exposures (Salingaros, 2018). Riley (2007) argued that language is more than a mere tool for communication. It serves as a vehicle for expressing ideologies, shaping collective perspectives, and reinforcing social strictures. It helps create cultural narratives, define identities, and establish societal boundaries. It does not simply reflect society; it actively affects and shapes it.

### 2.2 Role of language in sustaining family values

According to Karpava (2022), the importance of language cannot be overlooked, highlighting that each language is not only a cornerstone of cultural and personal identity but also a repository of distinct knowledge and expertise. Every language carriers its unique worldview. Preserving a language is closely associated with its speakers' self-esteem and cultural pride and the positive impact of strengthening family relations. Here, a study BY Nesteruk (2021) witnessed the importance of language among bilingual families residing in the United States. Data collected from 50 individuals was analyzed using phenomenological technique. Results revealed that the participants indicated an overall positive role of language in enhancing their cultural affiliation and family bonding. Despite the participants revealing some difficulties while residing in a multicultural society, they showed an overall positive role of language bridging the gaps between themselves, their ethnic and cultural identity, and familial relationships. As noted by Chen et al. (2012), families often use language to instill a sense of belonging and commitment among children toward their family. According to Ong (2021), transferring language to the coming generation is a complex sociolinguistic phenomenon. Family language policy indicates the extent to which they aim to transfer their cultural values to the young generation, which further determines the significance of language in a certain familial system.

### 2.3 Cultural transmission theory

Cultural transmission theory supports current research, proposing how certain factors affect social fabric (Mchitarjan and Reisenzein, 2014). Simply put, it indicates how certain cultural and social beliefs, convictions, and behaviors are transferred to the next generations, shaping contemporary society (Pagel, 2009). Based on this notion of cultural transmission theory, this research sees and proposes the role of language (Korneeva et al., 2019), particularly Arabic, in the United Arab Emirates. According to Chazy (2023), one of the reasons behind the increased importance and role of the Arabic language in Emirati society is the effect of the local government's efforts in supporting the use of the Arabic language on both domestic and official levels. Despite Emirati society being accompanied by multiculturalism, the importance of the Rabic language is determined by the fact that it is widely spoken and understood among all communities and nationalities. A study by Zakarneh et al. (2024) also affirmed the role of Arabic as a source of national cultural transmission in the UAE. Notably, some critics also highlight the effect of Western culture and the English language on Arabic language use in Emirati society. Still, its use among the young is prevalent (Manurung, 2018). Consistent with the previous argumentation, Raddawi and Meslem (2015) argue that language works as a both marker of identity and a representation of a particular community. It can influence changes in identity, nationalism, and ethnicity as a reflection of culture and society, assuming that the effect of the Arabic language on improving the resilience of the Emirati social and cultural system is effective (Zakarneh et al., 2024). Enfield (2014) considers cultural transmission through language a natural process carried out systematically from generation to generation. According to Enfield (2014), this cultural transmission through language takes years, as social and cultural values are linguistically transmitted in communicative contexts. This argument is particularly applicable in the current research where, besides social and cultural values, language is also proposed as a source of transmitting family values to the newer generations, further sustaining the social fabric of Emirati society through Arabic. Thus, given the empirical and theoretical literature, this study proposes the following hypotheses.

*H1*. Arabic Language Usage Positively Affect**s** Socio-Cultural Identity in the United Arab Emirates.
*H2*. Arabic language Usage positively affects Family Values in the United Arab Emirates.

## 3 Study methodology

### 3.1 Research design and data collection

This study adopted a quantitative design focusing on numerical data gathering from the respondents within a shorter time, offering greater generalizability of results (Hu and Chang, 2017). A structured questionnaire was further distributed, designed on five-point Likert scale (strongly agree, agree, neutral, disagree, and strongly disagree). Table 1 represents the study questionnaire and sources. After completing the data-gathering process, the collected responses were coded, and further analysis was conducted using SPSS and Structural Equation Modeling using Smart-PLS.

**Table 1:** Details of study questionnaire (items and sources).

**Variables**	**Items**	**Sources**
Arabic language usage	The Arabic language helps improve the sense of commitment in society.	(Smakman, 2012)
	The Arabic language encourages humility in society.	
	The Arabic language promotes a sense of unity among members of society in the UAE.	
	The Arabic language helps sustain social stability in Emirati society.	
	Community service participation is widely encouraged by the Arabic language.	
Socio-cultural identity	Speaking Arabic helps recognize socio-cultural value.	(Boyd et al., 2021)
	Arabic language enhances appreciation toward Emirati heritage.	
	The Arabic language improves social participation among the young generation.	
	Speaking Arabic helps people understand the importance of social laws.	
	The Arabic language enhances the sense of social responsibility among the young generation.	
Family values	The Arabic language improves family bonding among the young Emirati generation.	(Schwartz, 2008)
	The Arabic language helps Emirati youth to understand their family values better.	
	Speaking Arabic helps Emirati youth strengthen relationships with their ancestral heritage.	
	The Arabic language improves the sense of respect toward family among the young generation in the UAE.	
	Speaking Arabic at home helps me be closely linked to my family members.	

### 3.2 Sampling methods

The study surveyed 200 Emirati individuals from different sectors of society in the Emirates of Ajman, employing a simple random sampling approach. The sample size of 200 respondents was justified as the current research involved structural equation modeling, requiring a sample size ranging from 100 to 200 participants (DeliCe, 2018). Respondents were selected from different fields, including government, education, private sector, and others, ensuring a diverse representation of the Emirati population. Employing a simple random sampling approach improved the reliability and generalizability of the results, reducing potential bias in the conclusions. This technique is consistent with statistical theory, enabling strong inferences based on the field-collected data (van Haute, 2021). Notably, the study acquires a 100% response rate as all respondents submitted their responses accurately on time.

### 3.3 Research ethics

This study followed some primary ethics required in a study involving human participants (Dooly et al., 2017). First, informed consent (Ermine et al., 2004) was obtained from all the respondents. They were also informed that their precipitation would be voluntary and that they could withdraw from the process whenever they wanted without any further obligations. The respondents were also assured that their data would be used only for the current study and that the researchers would refrain from using it commercially.

## 4 Data analysis and results

Data analysis involved two phases. The first phase involved the calculation of respondents' demographics. The second phase included inferential analysis, including structural equation modeling. The relevant step included a two-step approach, including measurement model analysis, further leading to structural equation modeling.

### 4.1 Personal profile of respondents

Data revealed that 61 of respondents were males and 39% were females. Concerning age, 46.5% of respondents ranged from 18 to 22 years, 33.0% were 23 to 26 years old, 32.5% were 27 years old or above, and 13.5% were below 16 years. 59% of respondents indicated their qualification as “undergraduate,” 33.0% showed “graduate,” 2.5% indicated “post-graduation,” and 5.5% marked “Doctorate.” Finally, according to 69% of respondents, they reside in urban areas, 22.5% live in rural areas, and 8.5% marked “nomadic” as their area of residence. Table 2 represents the data regarding respondents' profiles.

**Table 2:** Personal profile of respondents.

**Variables**	**Constructs**	***N***	**%**
Gender	Male	122	61.0
	Female	78	39.0
Age	Below 16 years	27	13.5
	18–22 years	93	46.5
	23–26 years	66	33.0
	27 years or above	65	32.5
Qualification	Under graduate	118	59.0
	Graduate	66	33.0
	Post graduate	5	2.5
	Doctorate/PhD	11	5.5
Locality	Urban	138	69.0
	Rural	45	22.5
	Nomadic	17	8.5

### 4.2 Structural equation modeling (SEM)

After calculating the descriptives, structural equation modeling was conducted using a two-step approach. First, the validity and reliability of the measurement model/tool were tested to ensure its suitability for the structural model analysis (Flora et al., 2012) and the generalizability of results (Kiliç et al., 2020). First, Confirmatory Factor Analysis (CFA) is conducted, computing the factor loadings (FL), Average Variance Extracted Values (AVE), Cronbach Alpha (CA), and Composite Reliability (CR). Results of CFA indicated that most of the FL values exceed the threshold value of 0.5. AVE values also exceeded the threshold of 0.5 (Pesqué-Cela et al., 2021). Further, the Cronbach Alpha and Composite Reliability values were also found to have values exceeding the minimum threshold of 0.7 (Arifin and Yusoff, 2016). Overall, these results affirmed the reliability and validity of the measurement in the current study. Table 3 represents the results of the Confirmatory Factor Analysis (CFA).

**Table 3:** Confirmatory factor analysis (CFA).

**Variables**	**Items**	**Loadings**	**AVE**	**CA**	**CR**
Role of Arabic language	ALU1	0.702	0.594	0.744	0.829
	ALU2	0.579			
	ALU3	0.588			
	ALU4	0.634			
	ALU5	0.526			
Socio-cultural identity	SCV1	0.521	0.512	0.722	0.792
	SCV2	0.671			
	SCV3	0.615			
	SCV4	0.568			
	SCV5	0.588			
Family values	FAM1	0.697	0.559	0.753	0.784
	FAM2	0.741			
	FAM3	0.605			
	FAM4	0.501			

**Figure 1:**
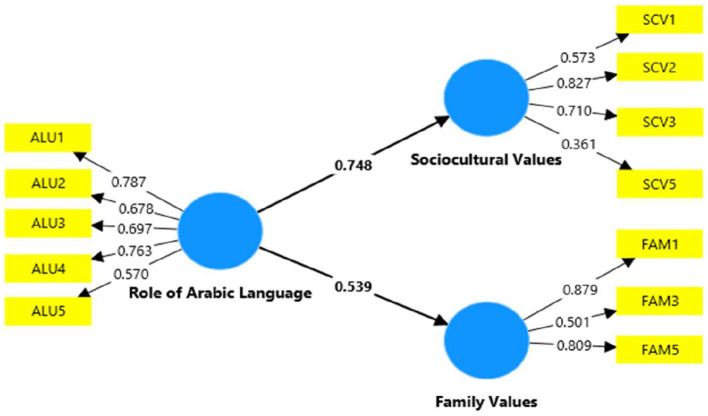
Final measurement model.

As some items, i.e., SCV4, FAM2, and FAM4, yielded loading values <0.5, they were further removed to test the suitability of the measurement model for the structural model analysis. In this regard, goodness of fit was tested as suggested by Schermelleh-Engel et al. (2003). Testing the goodness of fit indicated the Standardized Root Mean Square (SRMR) value of 0.0035 (<0.08) (Chwialkowski et al., 2018), Tucker and Lewis (TLI) value 0.956 (b/w 0–1), Non-Fit Index value 0.827 (b/w 0–1), and chi-square value −0.87601 (<3.0) (Demler et al., 2015). Overall, the analysis indicated a good fit for the structural model. Figure 1 shows the measurement model finalized after testing the goodness of fit.

Discriminant validity was further tested to ensure the extent to which study variables are distinct (Shiu et al., 2011). The Fornell-Lacrker criterion was employed (See Table 4). All the variables were distinct from each other, and the square roots of each AVE related to each variable (in Table 3) were also higher than the corresponding correlation values. These results affirmed the discriminant validity of the measurement tool in the current study.

**Table 4:** Discriminant validity (Fornell-Larcker criterion).

**Variables**	**Family values**	**Role of Arabic language**	**Socio-cultural values**
Family values	0.748		
Role of Arabic language	0.539	0.703	
Socio-cultural identity	0.598	0.348	0.642

Before testing the study hypotheses, the coefficient of determination *R*^2^, also known as *R* square analysis, was conducted. The relevant assessment was conducted to determine the predictive capability of the independent model, indicating its effect on dependent variables (Samartha and Kodikal, 2018). Table 5 represents the results of Coefficients of Determination *R*^2^. Analysis indicated a 55.9% variance in the variable “socio-cultural values” and a 59.0% variance in the “family values.” These results indicated a strong predictive power of the independent variable “Role of Arabic Language” in the current study.

**Table 5:** Coefficients of determination *R*^2^.

**Variables**	***R* ^2^**	**Strength**
Socio-cultural values	0.559	Strong
Family values	0.590	Strong

**Figure 2:**
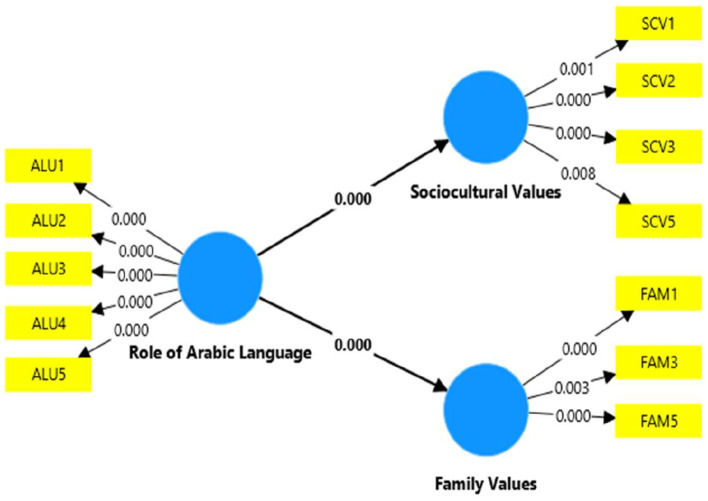
Results of structural model testing (*R*^2^ and path analysis).

Finally, the structural model was tested, accompanying path analysis and regression testing. According to Kelcey et al. (2021), despite regression being a distinct approach in cause and effect-based studies, path analysis provides an in-depth reflection of the hypothetical relationships between study variables. Considering the importance of path analysis, this study assessed two hypotheses. First, H1 proposed a positive role of Arabic Language Usage on Socio-Cultural Identity in the UAE. The first study hypotheses remained significant, with a *t*-value of 13.853 and a *p*-value of 0.000 (*p* < 0.05). The second hypothesis proposed a positive role of Arabic Language Usage on Family Values in the UAE and remained significant with a *t*-value of 9.539 and *p*-value of 0.000 (*p* < 0.05). Besides, the path between the Role of the Arabic Language and Socio-Cultural Identity remained strongest (0.748), while the path between the Role of the Arabic Language and Family Values was found to be comparatively weak (0.539). Table 6 represents the results of path analysis, and Figure 2 shows the final structural model, indicating all the significance values.

**Table 6:** Results of hypotheses testing (*R*^2^ and path analysis).

**Hyp**.	**Variables**	***M***	**SD**	**β**	***t*-statistics**	***p*-value**
*H1*.	Role of Arabic language → Socio-cultural identity	3.022	0.072	0.748	13.853	0.000
*H2*.	Socio-cultural values → Family values	3.650	0.078	0.539	9.539	0.000

## 5 Discussion

According to Castro et al. (2004), the evolution of linguistics and the use of language has ever increased due to the homogenization process. The acceptance of language as not merely a source of communication but also a means of transferring values to the next generation has even increased the importance of language in current social and cultural scenarios. Given the role of language assumed by cultural transmission theory, this study also witnessed the Arabic language serving similar functions. According to the respondents, the Arabic language plays a critical role in promoting a sense of commitment in society. They acknowledged that it encourages humility, reinforcing moral and ethical values among community members. They consented that the Arabic language promotes unity in Emirati society, sustaining cultural and traditional values. They also agreed that Arabic instills community service participation, enhancing collaboration and communication, reinforcing social cohesion and collective responsibility. Consistent with these results, Tong and Cheung (2011) also consider language a significant tool for expressing cultural identity while promoting shared understanding in culturally enriched societies. It reflects cultural differences through linguistic variations and plays a crucial role in shaping collective narratives, preserving history, and strengthening social relationships. Language also adapts to different cultural contexts, affecting communication patterns and social interactions.

Concerning the first hypothesis, “*H1. Arabic Language Usage Positively Affects Socio-Cultural Identity in the United Arab Emirates*,” the results indicated a strong agreement among the study respondents. They revealed that speaking Arabic is crucial in recognizing socio-cultural values and deepening appreciation for Emirati heritage. They also acknowledged that Arabic improves social participation among the younger generation, promoting a robust association with their cultural roots. Besides, they agreed that speaking Arabic helps Emirati youth understand the importance of social laws and promotes an increased sense of social responsibility. As noted by Hills and Atkins (2013), in societies like the United Arab Emirates, engaging young generations in cultural events, community gatherings, and other forms of social participation is well-served by the Arabic language. It establishes communication norms and shapes interpersonal relationships, contributing to the cultural fabric of a multicultural environment. This study has also highlighted the consistency with existing studies (Banks, 2011; Choi and Nunan, 2010; Kim, 2003), which show that language has influenced cultural and social identities within complex ethnic, religious, and socio-cultural systems. Studies suggest that social and cultural identity and stereotypes affect conversational patterns. Besides, language has also evolved in response to technological transformation and the rise of social media.

The second hypothesis, “*H2. Arabic Language Usage Positively Affects Family Values in the United Arab Emirates*,” also remained supported in the current study. The study respondents agreed that the Arabic language supports family relationships among the young Emirati generation and improves their understanding of family values. They highlighted that speaking Arabic helps them maintain a strong link with their ancestral heritage and promotes greater respect for family traditions. Also, they agreed that using Arabic at home improves their sense of closeness and belonging within their families. In line with the current study, Hopkyns (2016) argues that language is a critical source of preserving cultural heritage and family systems to express identity within different communities. For example, this study indicated how family relationships are strengthened through language, playing a unifying role by promoting intergroup solidarity and strengthening community bonds, also found in the existing literature (Hopkyns, 2020; Jose and Jacob, 2025). Thus, beyond its functional role, language actively contributes to social harmony and cohesion and enhances the national cultural identity in multicultural societies, including the United Arab Emirates. It is also notable that family interactions and relationships are important in maintaining diverse cultural identities (Ferguson et al., 2017). As the United Arab Emirates has a rich ethnic and cultural background, the Arabic language strongly affects how family members interact with each other and maintain a sense of commitment and belonging (Moussa-Inaty and Vega, 2013).

## 6 Study implications

The findings of this study have significant implications for cultural preservation, policymaking, and social cohesion in the UAE. The positive impact of Arabic language usage on socio-cultural identity and family values implies that language policies should continue reinforcing Arabic as a unifying force. This can guide educational institutions to integrate Arabic more deeply into the curriculum, ensuring that younger generations maintain strong cultural ties. Besides, the results suggest that initiatives promoting Arabic in public in the public sphere improve national identity and social stability. This study also highlights the necessity for media and digital platforms to support the Arabic language, countering the influence of foreign languages. Also, businesses and community organizations can use Arabic to strengthen local engagement and promote a sense of belonging. These results can inform future research on linguistics resources in multicultural societies, particularly in regions undergoing rapid globalization and technological advancements.

Similarly, policymakers, professionals, and cultural institutions can integrate Arabic to strengthen community engagement, improving the social fabric. This study also highlighted the significance of digital and technological advancements in promoting the Arabic language, filling the gap in the existing literature about the role of languages. These smiths can guide future studies on linguistic sustainability in evolving multicultural societies, mainly where external cultural forces influence national identity. Finally, this study emphasizes the importance of the Arabic language in shaping the UAE's socio-cultural landscape.

## 7 Conclusion

This study indicated that language, particularly Arabic, plays a crucial role in shaping societies by supporting social cohesions. It works as a bridge that connects individuals, ensuring the transmission of values and collective identity across generations. Arabic plays a fundamental role in shaping socio-cultural identity. It improves individuals' appreciation of Emirati heritage, promotes social participation, and reinforces a sense of responsibility and unity among the younger generation. Besides, it influences family relationships, deepening youth's understanding of ancestral heritage and promoting respect for family values. The study findings affirmed that the Arabic language is a key element in maintaining the stability of Emirati society, promoting social commitment and inclusivity. Overall, this study highlighted the critical role of language in preserving cultural identity, promoting social harmony, refining family values, and ensuring the sustainability of Emirati traditions in an evolving world.

## 8 Limitations

This study has some limitations that are crucial to be highlighted. First, this study has a limitation regarding geographical generalizability. As it focuses only on one Emirate of the UAE, Ajman, the results cannot be generalized to other regions. The second limitation includes the use of a single methodological approach. Using only quantitative design further narrows down its scope. Finally, the third limitation involves focusing on an overall social and cultural identity while overlooking the role and effect of language on individual identity, which is also considerable. Future researchers can counter these limitations, adopt further approaches, and conduct their studies in different regions to delimit this scope.

## Data Availability

The raw data supporting the conclusions of this article will be made available by the authors, without undue reservation.
